# Phylodynamics of the HIV-1 Epidemic in Cuba

**DOI:** 10.1371/journal.pone.0072448

**Published:** 2013-09-09

**Authors:** Edson Delatorre, Gonzalo Bello

**Affiliations:** Laboratório de AIDS e Imunologia Molecular, Instituto Oswaldo Cruz, FIOCRUZ, Rio de Janeiro, Brazil; Institut Pasteur of Shanghai, Chinese Academy of Sciences, China

## Abstract

Previous studies have shown that the HIV-1 epidemic in Cuba displayed a complex molecular epidemiologic profile with circulation of several subtypes and circulating recombinant forms (CRF); but the evolutionary and population history of those viral variants remains unknown. HIV-1 *pol* sequences of the most prevalent Cuban lineages (subtypes B, C and G, CRF18_cpx, CRF19_cpx, and CRFs20/23/24_BG) isolated between 1999 and 2011 were analyzed. Maximum-likelihood analyses revealed multiple introductions of subtype B (*n*≥66), subtype C (*n*≥10), subtype G (*n*≥8) and CRF18_cpx (*n*≥2) viruses in Cuba. The bulk of HIV-1 infections in this country, however, was caused by dissemination of a few founder strains probably introduced from North America/Europe (clades B_CU-I_ and B_CU-II_), east Africa (clade C_CU-I_) and central Africa (clades G_CU_, CRF18_CU_ and CRF19_CU_), or locally generated (clades CRFs20/23/24_BG). Bayesian-coalescent analyses show that the major HIV-1 founder strains were introduced into Cuba during 1985–1995; whereas the CRFs_BG strains emerged in the second half of the 1990s. Most HIV-1 Cuban clades appear to have experienced an initial period of fast exponential spread during the 1990s and early 2000s, followed by a more recent decline in growth rate. The median initial growth rate of HIV-1 Cuban clades ranged from 0.4 year^−1^ to 1.6 year^−1^. Thus, the HIV-1 epidemic in Cuba has been a result of the successful introduction of a few viral strains that began to circulate at a rather late time of the AIDS pandemic, but then were rapidly disseminated through local transmission networks.

## Introduction

The global dissemination of the Human immunodeficiency virus type 1 (HIV-1) group M clade during the second half of the twentieth century has resulted in the generation of a diverse collection of genetic variants classified into subtypes, sub-subtypes, circulating recombinant forms (CRFs) and unique recombinants forms (URFs). The HIV-1 epidemic in the Americas is typically dominated by subtype B clade, although substantial proportions (≥20%) of non-B subtype genetic forms are observed in Argentina, Brazil, Cuba and Uruguay [1].

Cuba displayed a unique HIV-1 molecular epidemiologic profile in the Americas characterized by the co-circulation of several subtypes (A1, B, C, F1, G, H and J), CRFs and URFs. Subtype B is the most prevalent variant (∼33–40%), followed by CRF19_cpx (∼20–28%), CRFs20/23/24_BG (∼12–20%) CRF18_cpx (∼7–10%), subtype C (∼3–10%), and subtype G (∼2–7%) [2], [3], [4], [5], [6], [7]. It has been proposed that the presence of numerous Cuban military and civilian personnel in several sub-Saharan African countries, and particularly those stationed in Angola and neighboring countries between 1975 and 1991, have contributed to the introduction of multiple non-B HIV-1 subtypes into Cuba [2]. Some HIV-1 recombinants including CRF18_cpx and CRF19_cpx were probably also imported into Cuba directly from central Africa, since the parental viruses of these complex genetic forms were only detected in that African region [8], [9]. Indeed, a few cases of CRF18_cpx and CRF19_cpx like viruses have been confirmed in Angola [10], [11], Democratic Republic of Congo (DRC) [12], [13], Republic of Congo [14], [15], Central African Republic [16], and Cameroon [17], [18], [19]. Other HIV-1 recombinants including all three CRFs_BG, however, were probably generated locally by recombination between subtypes B and G already circulating in Cuba [20].

According to this model, most non-B subtype HIV-1 variants circulating in Cuba were probably introduced or locally generated after 1975. Despite the extensive knowledge about the molecular epidemiology of HIV-1 variants, the time-scale and epidemic history of most prevalent HIV-1 clades circulating in Cuba remains to be elucidated. In this study, we used a Bayesian coalescent-based method and a comprehensive data set of HIV-1 subtype B (*n* = 322), and non-B subtypes (*n* = 420) *pol* sequences of Cuban origin isolated between 1999 and 2011, to date the origin and reconstruct the demographic history of major HIV-1 variants circulating in Cuba.

## Materials and Methods

### HIV-1 Cuban sequence datasets

We downloaded all HIV-1 Cuban sequences covering the entire protease and partial reverse transcriptase (PR/RT) regions of the *pol* gene (nt 2253–3272 relative to HXB2 clone) classified as subtypes B (*n* = 322), C (*n* = 49), G (*n* = 35), CRF18_cpx (*n* = 71), CRF19_cpx (*n* = 167), and CRFs20/23/24_BG (*n* = 118) that were available at the Los Alamos HIV Sequence Database (www.hiv.lanl.gov) by March 2013. HIV-1 *pol* sequences were retrieved from both antiretroviral therapy naïve and HAART treated patients from different Cuban regions between 1999 and 2011, as described in previous studies [2], [3], [4], [5], [6]. Sequences were aligned using the CLUSTAL X program [21]. To avoid any bias on the phylogenetic reconstructions, all sites with major antiretroviral drug resistance mutations in PR (30, 32, 46, 47, 48, 50, 54, 76, 82, 84, 88 and 90) or RT (41, 65, 67, 69, 70, 74, 100, 101, 103, 106, 115, 138, 151, 181, 184, 188, 190, 210, 215, 219 and 230) detected in at least two sequences were excluded from each alignment. All alignments are available from the authors upon request.

### HIV-1 reference datasets

HIV-1 Cuban sequences were combined with reference sequences of diverse origin that matched the selected genomic region and were available at the Los Alamos HIV Sequence Database. Subtype B Cuban sequences were aligned with reference sequences representative of the viral diversity in US (*n* = 525), France (*n* = 348) and the Caribbean (*n* = 417) (Table S1). Subtype C Cuban sequences were aligned with representative sequences from central (*n* = 53), eastern (*n* = 330) and southern (*n* = 545) African regions (Table S2). The HIV-1 subtype G Cuban sequences were combined with all available subtype G sequences of African origin (*n* = 437) (Table S3). The CRF19_cpx Cuban sequences were aligned with all available CRF19_cpx sequences from other countries (*n* = 3) and subtype D sequences of African origin (*n* = 1,112) (Table S4). Finally, the HIV-1 CRF18_cpx and CRFs_BG Cuban sequences were combined with all available CRF18_cpx (*n* = 15) and CRFs20/23/24_BG (*n* = 7) sequences from other countries (Tables S4 and S5).

### Subtype assignment

The subtype assignment and recombinant structure of all sequences here included was confirmed by: REGA HIV subtyping tool v.2 [22]; Bootscanning with Simplot software v3.5.1 [23] and Maximum Likelihood (ML) phylogenetic analysis. In bootscan analyses, supporting branching with reference sequences from all HIV-1 group M subtypes were determined in Neighbor-Joining trees based on 100 re-samplings, within a 250 bp window moving in steps of 10 bases. ML phylogenetic trees were inferred under the best nucleotide substitution model selected using the jModeltest program [24] (Table S6). The ML tree was reconstructed with the PhyML program [25] using an online web server [26]. Heuristic tree search was performed using the SPR branch-swapping algorithm and the reliability of the obtained topology was estimated with the approximate likelihood-ratio test (*aLRT*) [27] based on the Shimodaira-Hasegawa-like procedure. The ML trees were visualized using the FigTree v1.4.0 program [28]. All HIV-1 sequences displaying incorrect clade assignment and/or frameshift mutations were excluded from the study, with the exception of four CRF23_BG sequences that were reclassified as CRF20_BG (GenBank accession numbers FJ481689 and FJ585687) and CRF24_BG (GenBank accession numbers JQ585465 and FJ481688).

### Reconstruction of evolutionary and demographic history

The evolutionary rate (*μ*, nucleotide substitutions per site per year, subst./site/year), the age of the most recent common ancestor (*T*_mrca,_ years), and the mode and rate (r, years^−1^) of population growth of different Cuban HIV-1 clades were jointly estimated using the Bayesian Markov Chain Monte Carlo (MCMC) approach as implemented in BEAST v1.7.5 [29], [30]. Analyses were performed using the best nucleotide substitution model (Table S6) and an uncorrelated Lognormal relaxed molecular clock model [31]. A Bayesian Skyline coalescent tree prior [32] was first used to estimate *μ*, the *T*_mrca_, and the change in effective population size through time. Estimates of the population growth rate were subsequently obtained using a logistic growth coalescent tree prior that was the model pointed out by the Bayesian Skyline plot and that also provided the best fit to the demographic signal contained in most datasets. Comparison between demographic models was performed using the log marginal likelihood (ML) estimation based on path sampling (PS) and stepping-stone sampling (SS) methods [33]. MCMC chains were run for 10–50×10^6^ generations. Adequate chain mixing and uncertainty in parameter estimates were assessed by calculating the effective sample size (ESS) and the 95% Highest Probability Density (HPD) values respectively, after excluding an initial 10% using the TRACER v1.5 program [34].

## Results

### Identification of major HIV-1 Cuban clades

**Figure 1:**
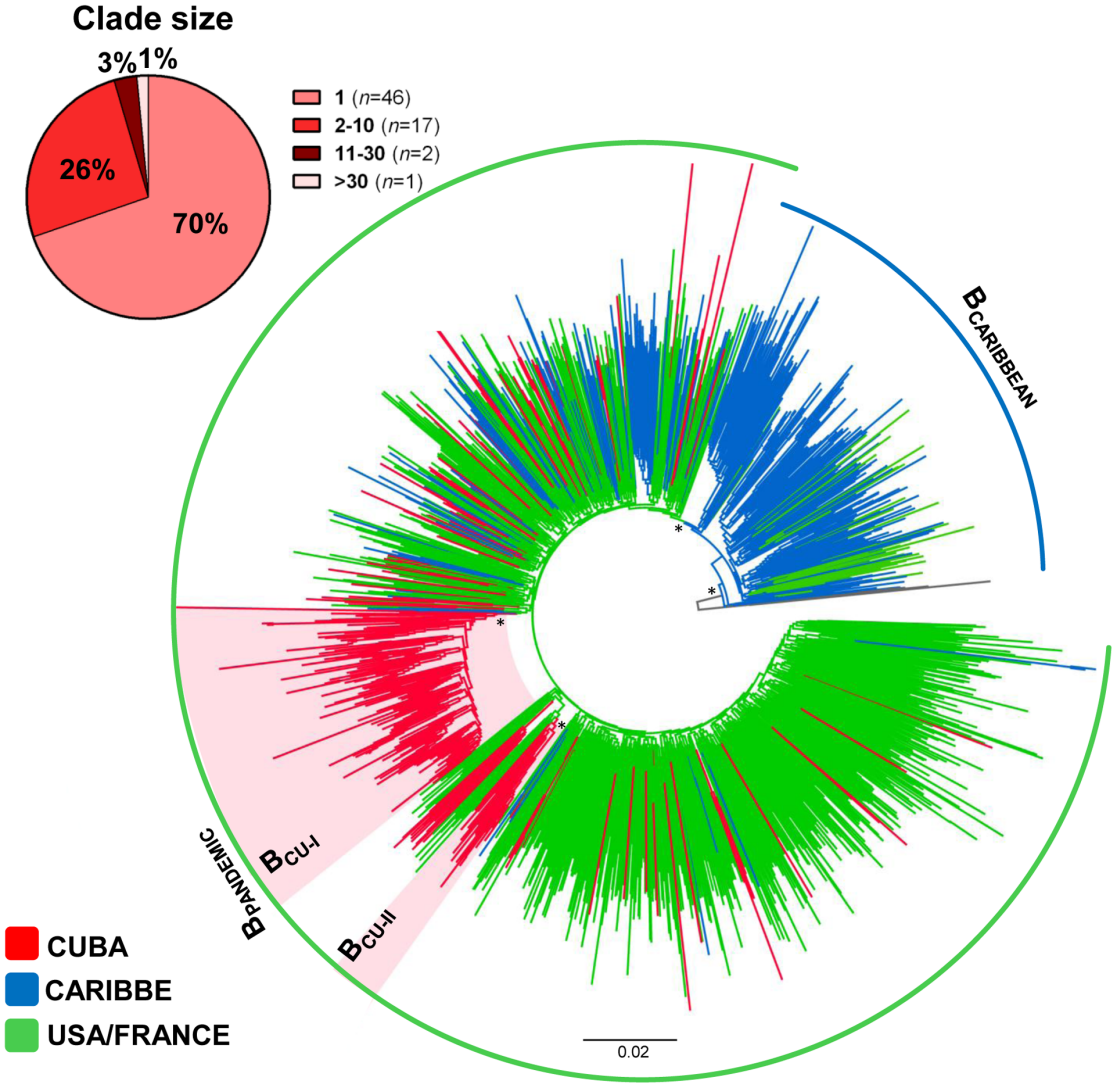
ML phylogenetic tree of HIV-1 subtype B *pol* (∼1000 pb) sequences circulating in Cuba (*n* = 322), US (*n* = 525), France (*n* = 348), and other Caribbean countries (*n* = 418). The branches are colored according to the origin of each sequence, as indicated at the legend (bottom left). The circular brackets highlight the position of the pandemic (B_PANDEMIC_, green line) and non-pandemic (B_CARIBBEAN_, blue line) HIV-1 subtype B clades. Shaded boxes highlight the position of the two major HIV-1 subtype B Cuban clades (B_CU-I_ and B_CU-II_). The number of Cuban sequences distributed accordingly to the clade size is shown (top left). Key nodes with *a*LRT support values >0.80 (*) and ≥0.90 (**) are indicated. The tree was rooted using HIV-1 subtype D reference sequences (gray branches). The branch lengths are drawn to scale with the bar at the bottom indicating nucleotide substitutions per site.

The ML analysis of HIV-1 subtypes B, C and G sequences from Cuba and other countries from the Americas, Europe and Africa revealed that most Cuban strains branched in well-supported country-specific sub-clades. Of the 322 HIV-1 subtype B Cuban sequences analyzed, 180 (56%) formed a large country-specific monophyletic sub-clade (B_CU-I_, *aLRT* = 0.81), 44 (14%) branched in two clusters of medium size (15<*n*<30), 52 (16%) formed clusters of small size (*n*≤10), and the remaining 46 (14%) represented non-clustered sequences (Fig. 1). Of note, all subtype B Cuban sequences branched in a large B_PANDEMIC_ monophyletic cluster (*aLRT* = 0.80) together with most subtype B sequences from US (92%) and all sequences from France (100%); whereas most non-Cuban Caribbean sequences (60%) occupy the deepest branches within B clade (Fig. 1).

**Figure 2:**
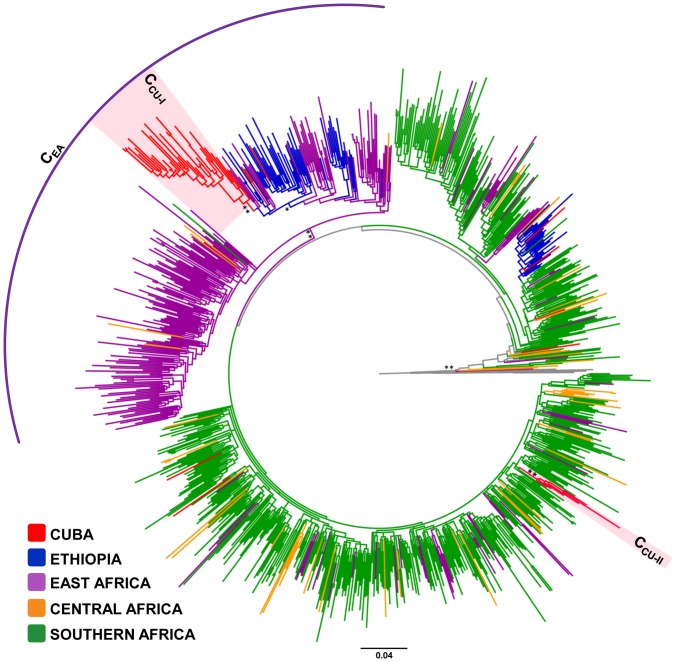
ML phylogenetic tree of HIV-1 subtype C *pol* (∼1000 pb) sequences circulating in Cuba (*n* = 49), and in central (*n* = 53), eastern (*n* = 330) and southern (*n* = 545) African countries. Branches are colored according to the origin of each sequence, as indicated at the legend (bottom left). The circular bracket highlights the position of the subtype C east African clade (C_EA_). Shaded boxes highlight the position of the two major HIV-1 subtype C Cuban clades (C_CU-I_ and C_CU-II_). Key nodes with *a*LRT support values >0.80 (*) and ≥0.90 (**) are indicated. The tree was rooted using HIV-1 subtype A1 and D reference sequences (gray branches). The branch lengths are drawn to scale with the bar at the bottom indicating nucleotide substitutions per site.

Of the 49 HIV-1 subtype C Cuban sequences analyzed, 34 (69%) branched in a single monophyletic sub-cluster (C_CU-I_, *aLRT* = 0.94), six (12%) branched in a second well supported minor clade (C_CU-II_, *aLRT* = 0.98), and the remaining nine (18%) represented non-clustered sporadic lineages (Fig. 2). The major clade C_CU-I_ was nested within Ethiopian sequences that belongs to the previously called C_EA_ clade [35], a viral lineage characteristic of the east African region (Fig. 2). The minor clade C_CU-II_, by contrast, was nested within subtype C sequences from southern Africa (Fig. 2). Non-clustered Cuban sequences were scattered among strains from Ethiopia and southern African countries.

**Figure 3:**
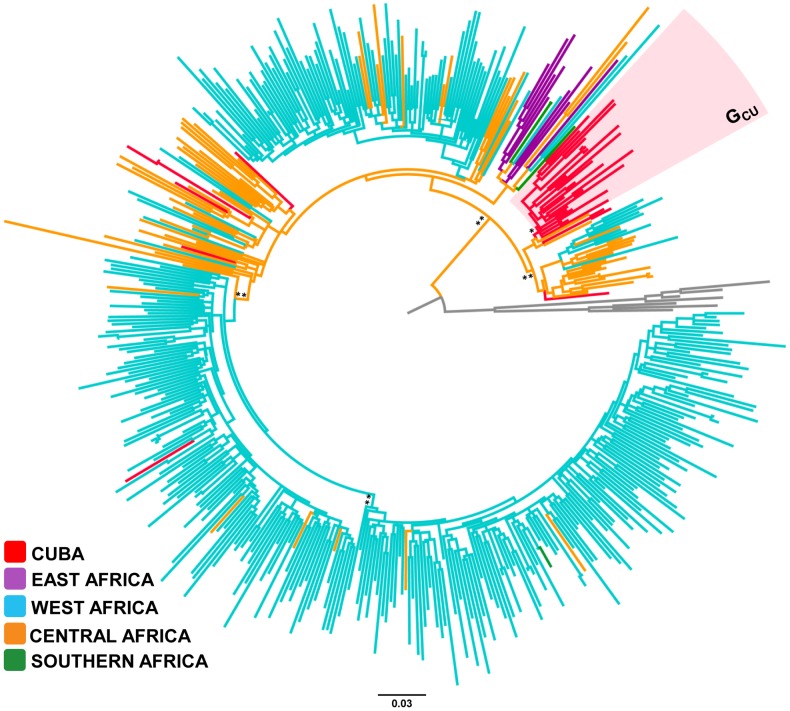
ML phylogenetic tree of HIV-1 subtype G *pol* (∼1000 pb) sequences circulating in Cuba (*n* = 35), and in central (*n* = 71), western (*n* = 366), eastern (*n* = 10) and southern (*n* = 3) African countries. Branches are colored according to the origin of each sequence, as indicated at the legend (bottom left). Shaded boxes highlight the position of the major HIV-1 subtype G Cuban clade (G_CU_). Key nodes with *a*LRT support values >0.80 (*) and ≥0.90 (**) are indicated. The tree was rooted using HIV-1 subtype A1 and B reference sequences (gray branches). The branch lengths are drawn to scale with the bar at the bottom indicating nucleotide substitutions per site.

Of the 35 HIV-1 subtype G Cuba sequences analyzed, 26 (74%) branched in a single monophyletic sub-cluster (G_CU_, *aLRT* = 0.87) and the remaining nine (26%) represented sporadic lineages of one or two sequences. Although most subtype G African strains included in our analysis were from the western region (*n* = 366, 84%), the clade G_CU_ and most sporadic subtype G Cuban lineages were nested among basal sequences from the central African region (Angola, DRC and Cameroon) (Fig. 3). There was only one Cuban sequence that branched within a major African subtype G sub-clade mainly composed by sequences from Nigeria.

**Figure 4:**
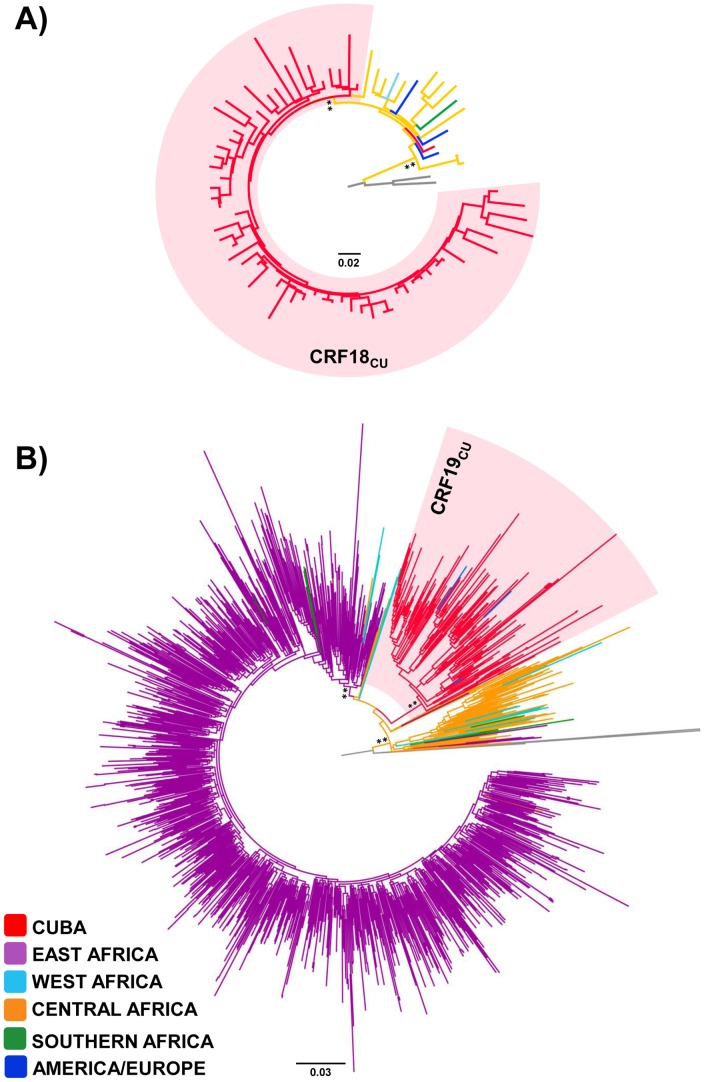
ML phylogenetic trees of HIV-1 CRFs_cpx *pol* (∼1000 pb) sequences. A) HIV-1 CRF18_cpx from Cuba (*n* = 62), were combined with those isolated in African (*n* = 12), American (*n* = 1) and European (*n* = 2) countries. The tree was rooted using HIV-1 subtype G reference sequences (black branches). B) HIV-1 CRF19_cpx sequences from Cuba (*n* = 160) and European countries (*n* = 3) were combined with subtype D sequences of African origin (*n* = 1,112). Branches are colored according to the origin of each sequence, as indicated at the legend (bottom left). Shaded boxes highlight the position of the major HIV-1 CRF18_cpx (CRF18_CU_) and CRF19_cpx (CRF19_CU_) Key nodes with *a*LRT support values >0.80 (*) and ≥0.90 (**) are indicated. The branch lengths are drawn to scale with the bar at the bottom indicating nucleotide substitutions per site.

Test the monophyletic origin of the HIV-1 CRFs_cpx Cuban sequences was very much complicated because the scarcity of CRF18_cpx (*n* = 12) and the absence of CRF19_cpx *pol* sequences of African origin available in public databases. Because CRF19_cpx is subtype D in the *pol* fragment analyzed, we decided to include all available subtype D *pol* sequences of African origin in our analysis. ML analysis revealed that all (except one) CRF18_cpx and all CRF19_cpx sequences from Cuba branched in highly supported (*aLRT*≥0.90) monophyletic sub-clusters (CRF18_CU_ and CRF19_CU_) that were nested within CRF18_cpx and subtype D *pol* sequences of central African origin, respectively (Fig. 4). The few CRF18_cpx isolated in Europe (*n* = 2) and South America (*n* = 1) were intermixed among basal African strains; whereas all CRF19_cpx detected in Europe (*n* = 3) branched within the clade CRF19_CU_ (Fig. 4).

**Figure 5:**
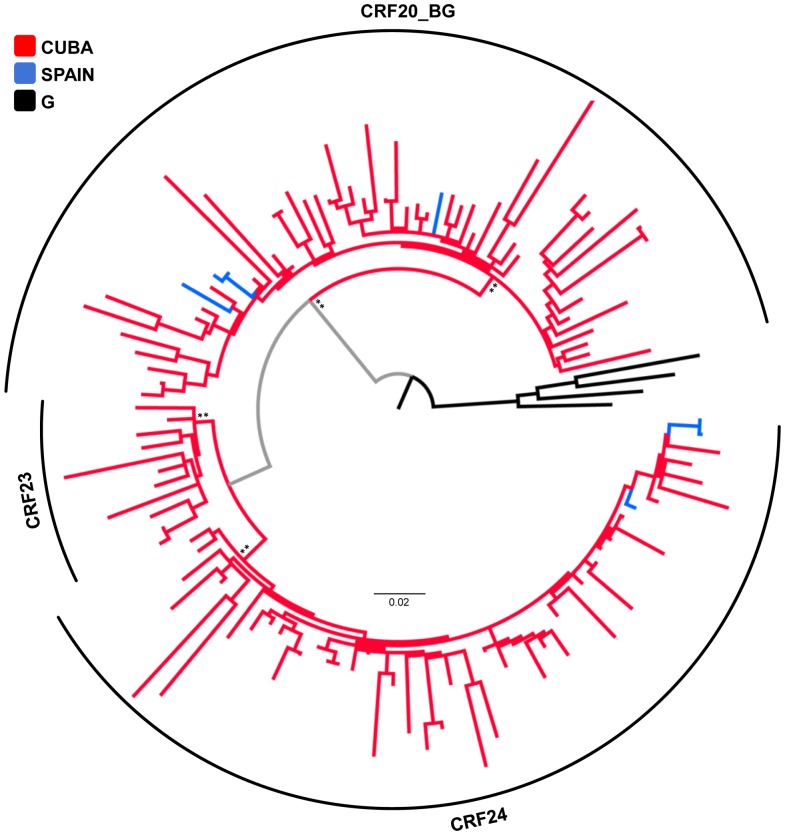
Ml phylogenetic tree of HIV-1 CRFs20/23/24_BG *pol* (∼1000 pb) sequences circulating in Cuba (*n* = 118) and Spain (*n* = 7). Branches are colored according to the origin of each sequence, as indicated at the legend (top left). The circular brackets highlight the distribution of the three CRFs_BG clades. The tree was rooted using HIV-1 subtype G reference sequences (black branches). Key nodes with *a*LRT support values >0.80 (*) and ≥0.90 (**) are indicated. The branch lengths are drawn to scale with the bar at the bottom indicating nucleotide substitutions per site.

As expected, the CRFs20/23/24_BG Cuban sequences formed three well-supported (*aLRT*≥0.90) monophyletic lineages (Fig. 5). The few CRF20_BG (*n* = 4) and CRF24_BG (*n* = 3) sequences isolated outside Cuba (Spain and Greece) were intermixed among Cuban strains (Fig. 5); thus supporting a Cuban origin for all those European sequences.

### Time scale of major HIV-1 Cuban clades

Bayesian MCMC analyses under a relaxed molecular clock model were used to estimate the substitution rate and T_MRCA_ of all HIV-1 Cuban clades with a minimum size of 25 sequences. A few subtype B (*n* = 4) and CRF19_cpx (*n* = 2) sequences with anomalously long branches in the phylogenetic tree, were excluded. The final number of HIV-1 Cuban sequences included in the evolutionary analyses is shown in Table 1. The median estimated evolutionary rates for the *pol* region of the different HIV-1 clades were roughly similar, ranging from 2.0×10^−3^ subst./site/year (G_CU_ clade) to 3.4×10^−3^ subst./site/year (CRF19_CU_ clade), with a considerable overlap of the 95% HPD intervals (Table 1). The coefficient of rate variation was higher than zero for all HIV-1 datasets analyzed (Table 1), thus supporting the use of a relaxed molecular clock model to reconstruct the time-scale of major HIV-1 Cuban lineages.

**Table 1:** Evolutionary rate and time-scale of major HIV-1 Cuban clades.

HIV-1 clade	*N*	Sampling interval	*μ* (subst./site/year)	Coefficient of variation	T_MRCA_
B_CU-I_	176	2003–2011	3.0×10^−3^	0.30	1992
			(2.4×10^−3^–3.6×10^−3^)	(0.21–0.39)	(1988–1994)
B_CU-II_	27	1999–2011	2.4×10^−3^	0.25	1991
			(1.6×10^−3^–3.2×10^−3^)	(0.01–0.45)	(1986–1994)
C_CU_	34	2003–2011	2.8×10^−3^	0.41	1994
			(2.0×10^−3^–3.8×10^−3^)	(0.19–0.65)	(1990–1998)
G_CU_	26	1999–2011	2.0×10^−3^	0.56	1988
			(1.0×10^−3^–3.3×10^−3^)	(0.36–0.81)	(1976–1995)
CRF18_CU_	61	1999–2011	2.6×10^−3^	0.40	1992
			(1.9×10^−3^–3.5×10^−3^)	(0.25–0.59)	(1987–1996)
CRF19_CU_	158	1999–2011	3.4×10^−3^	0.38	1987
			(2.9×10^−3^–4.0×10^−3^)	(0.30–0.47)	(1983–1991)
CRF20/23/24_BG	117	1999–2011	2.6×10^−3^	0.35	1991
			(2.1×10^−3^–3.1×10^−3^)*	(0.25–0.45)*	(1986–1994)*
CRF20_BG	56	1999–2011	2.6×10^−3^	0.35	1996
			(2.1×10^−3^–3.1×10^−3^)*	(0.25–0.45)*	(1994–1998)*
			2.4×10^−3^	0.27	1996
			(1.8×10^−3^–3.0×10^−3^)	(0.10–0.44)	(1994–1998)
CRF23_BG	11	2003–2011	2.6×10^−3^	0.35	1998
			(2.1×10^−3^–3.1×10^−3^)*	(0.25–0.45)*	(1996–2000)*
CRF24_BG	50	2003–2011	2.6×10^−3^	0.35	1997
			(2.1×10^−3^–3.1×10^−3^)*	(0.25–0.45)*	(1996–1999)*
			2.2×10^−3^	0.36	1998
			(1.6×10^−3^–2.8×10^−3^)	(0.19–0.54)	(1996–2000)

* Estimates obtained from the combined CRF20/23/24_BG data set.

The median T_MRCA_ of those HIV-1 clades imported into Cuba range between 1987 (CRF19_CU_) and 1994 (C_CU-I_); whereas the median T_MRCA_ of those CRF_BG variants locally generated varied between 1996 and 1998 (Table 1). The T_MRCA_ of CRF20_BG and CRF24_BG clades estimated from the single CRF datasets were almost identical to those estimated from the combined CRFs20/23/24_BG data set (Table 1), indicating that all Cuban CRFs_BG evolved at quite similar rates. A previous study [20], proposed that Cuban CRF_BG viruses derive from a common recombinant ancestor generated by recombination between clade G_CU_ and the second most prevalent subtype B clade (B_CU-II_) (Fig. 1). The analysis of the combined CRFs20/23/24_BG data set allows us to estimate the median T_MRCA_ of that putative BG recombinant ancestor at 1991, roughly coinciding with the estimated T_MRCA_ of the parental clades G_CU_ and B_CU-II_ (Table 1).

### Demographic history of major HIV-1 Cuban clades

**Figure 6:**
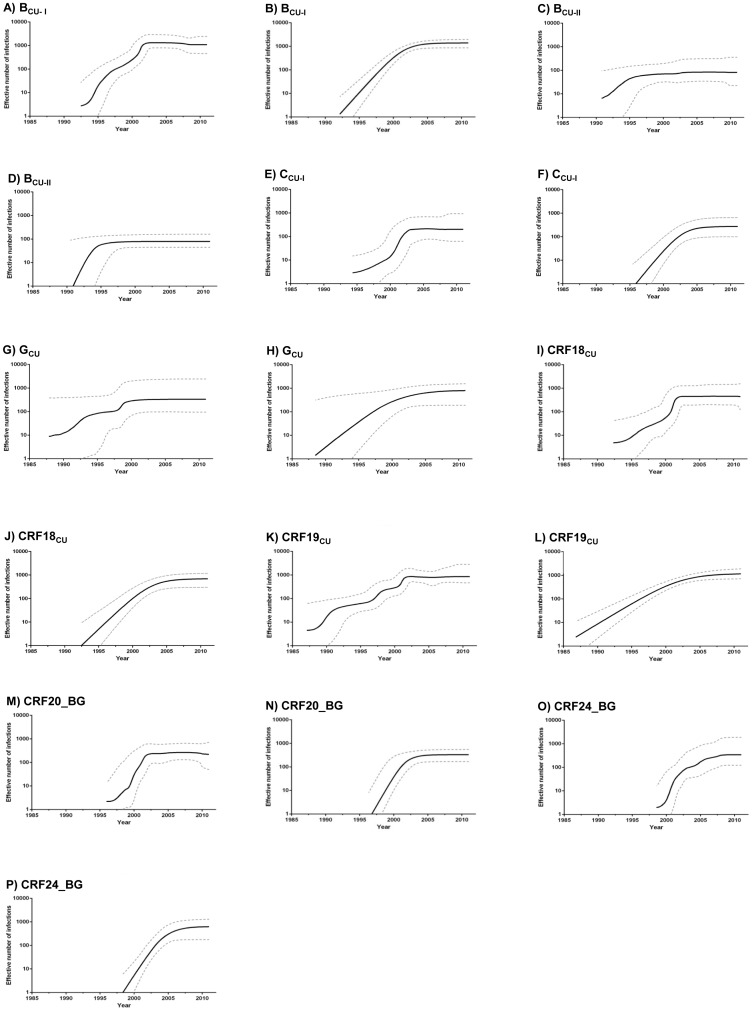
Demographic history of the major HIV-1 Cuban clades. Effective number of infections through time estimated using both Bayesian skyline (A, C, E, G, I, K, M and O) and logistic growth (B, D, F, H, J, L, N and P) coalescent models are shown for each of the eight HIV-1 Cuban clades analyzed. Median estimates of the effective number of infections (solid line) and 95% HPD intervals of the estimates (dashed lines) are shown in each graphic. The vertical axes represent the estimated effective number of infections on a logarithmic scale. Time scale is in calendar years.

The Bayesian skyline plot (BSP) analyses suggest that all HIV-1 Cuban clades experienced an initial phase of fast exponential growth followed by a more recent decline in growth rate (Fig. 6). The growth rate of most HIV-1 Cuban clades seems to start to decrease around the early 2000s; except for clades B_CU-II_ and CRF24_BG that seem to stabilize at earlier (before 2000) and later (after 2005) time points, respectively. The BSP analyses also suggests that the coalescent model of logistic population growth fits the demographic information contained in all HIV-1 Cuban data sets better than the exponential one.

**Figure 7:**
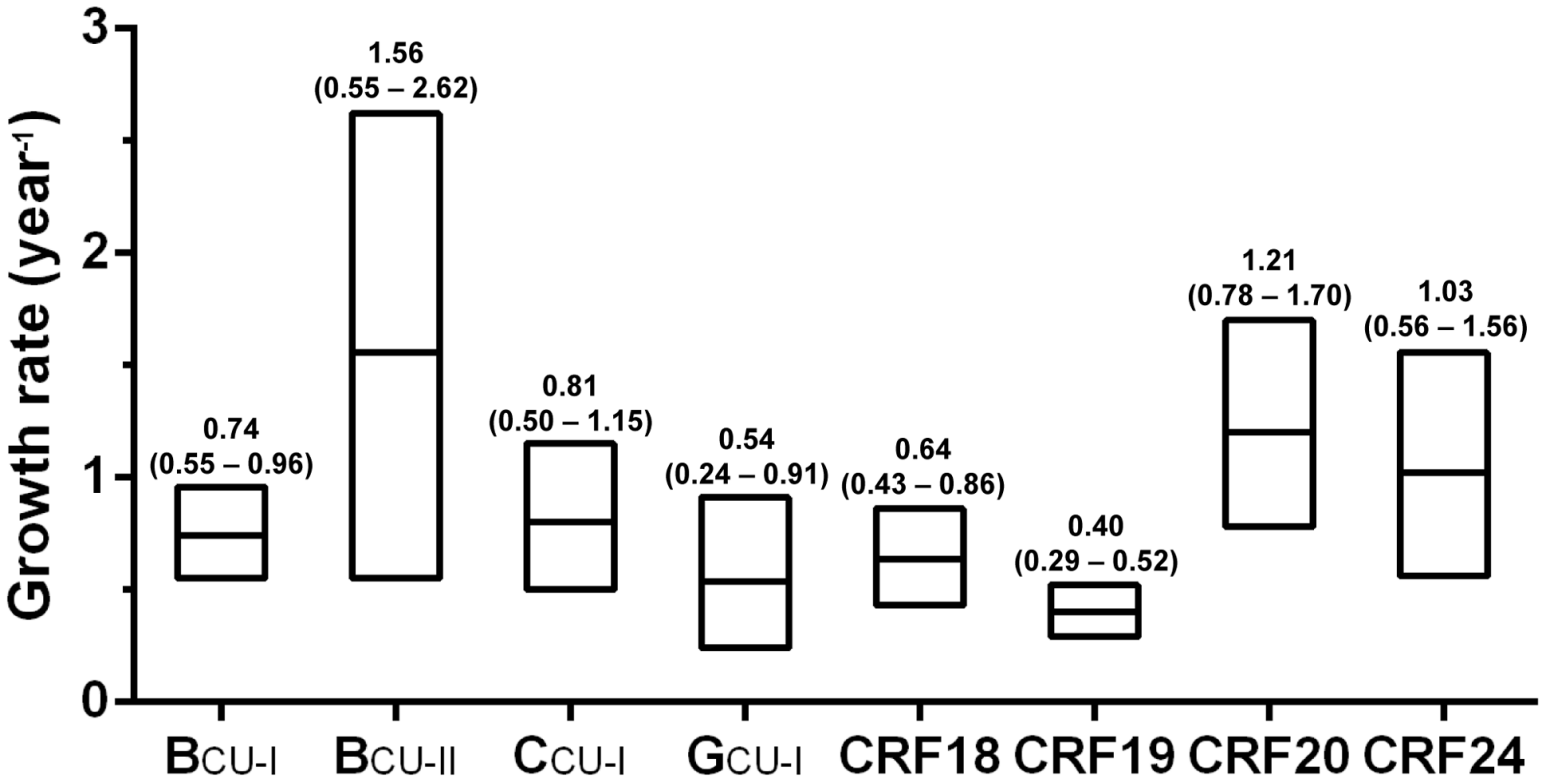
Coalescent estimates of epidemic growth rate of the major HIV-1 Cuban clades. The box plots and the numbers above represent the median growth rates (years^−1^) and the 95% HPD intervals of the posterior distributions estimated under the logistic growth coalescent model for each of the eight HIV-1 Cuban clades analyzed.

To test this, log ML for the logistic and exponential growth models were calculated using both PS and SS methods. The model of logistic population growth was strongly supported over the exponential one for most HIV-1 Cuban clades (log BF>3), with exception of G_CU_ and CRF24_BG for which only a weak support was obtained (log BF = 0.9–1.0) (Table 2). Such a low BF support to the logistic growth model could be explained by the low number of sequences in clade G_CU_ (*n* = 26) and the very recent stabilization of clade CRF24_BG (after 2005). Moreover, the overall time-scale and demographic pattern obtained from both BSP and logistic growth coalescent tree priors were very similar for all HIV-1 Cuban clades (Fig. 6). According to the logistic model, the median initial growth rates of HIV-1 Cuban clades range between 0.40 year^−1^ (CRF19_CU_) to 1.57 year^−1^ (B_CU-II_) with some overlap of the 95% HPD intervals for most lineages, except between CRF19_CU_ and clades B_CU-I_, B_CU-II_, C_CU-I_, CRF20_BG and CRF24_BG (Fig. 7).

**Table 2:** Best fit demographic model for major HIV-1 Cuban clades.

Dataset	PS			SS		
	Log ML LG	Log ML EG	Log BF (LG vs EG)	Log ML LG	Log ML EG	Log BF (LG vs EG)
B_CUBA I_	−12891.71	−12935.39	43.68	−12894.56	−12939.47	43.91
B_CUBA II_	−3563.92	−3568.51	4.59	−3563.99	−3568.71	4.72
C_CUBA_	−4114.63	−4118.50	3.87	−4114.74	−4118.72	3.98
G_CUBA_	−4196.64	−4197.66	1.02	−4196.87	−4197.73	0.86
CRF18	−6109.29	−6118.91	9.62	−6109.54	6119.01	9.47
CRF19	−14552.57	−14564.21	11.64	−14553.91	−14565.09	11.18
CRF20	−5083.74	−5100.80	17.06	−5083.91	−5101.05	17.14
CRF24	−4159.40	−4160.43	1.03	−4159.81	−4160.70	0.89

Log marginal likelihood (ML) estimates for logistic growth (LG) and exponential growth (EG) demographic models obtained using the path sampling (PS) and stepping stone sampling (SS) methods. The Log Bayes factor (BF) is the difference of the Log ML between of alternative (H1 = LG) and null (H0 = EG) models. Log BFs>1 indicates that model H1 is more strongly supported by the data than model H0.

## Discussion

The Cuban HIV epidemic is unique in the Americas because of the exceptionally low HIV prevalence, estimated at 0.20% in adults in 2011 [36], and the unusually high HIV-1 genetic diversity with circulation of subtype B and several non-B subtypes [2], [3], [4], [5], [6], [7]. Our study indicates that most HIV-1 infections in Cuba derived from the dissemination of a few founder viruses that were either introduced from the Americas/Europe (subtype B) and Africa (subtype C, subtype G, CRF18_cpx and CRF19_cpx) or were locally generated (CRFs20/23/24_BG).

The most accepted model of worldwide HIV-1 subtype B dissemination suggests that the virus moved from Haiti to other Caribbean islands and to the United States (US), and then from the US to the rest of the world establishing a “B_PANDEMIC_” clade [37]. The phylogenetic analysis here performed revealed multiple (*n*≥66) introductions of HIV-1 B_PANDEMIC_ strains in Cuba, although the bulk of the subtype B epidemic in this country resulted from the dissemination of only a few clades. The two most prevalent clades B_CU-I_ and B_CU-II_ comprises about 55% and 8% of all subtype B sequences from Cuba here included, respectively. We estimate that these clades most probably emerged in Cuba in the early 1990s, much later than the estimated origin of subtype B epidemics in Haiti and the US (1960–1970) [37], [38], [39]. The estimated T_MRCA_ of clades B_CU-I_ and B_CU-II_ coincides with a crisis in the Cuban economy caused by the collapse of the Soviet Union in 1991 that precipitated important investments in the tourist industry and a sharp increase in the number of tourist mostly from North America and Europe [40], regions with a widespread circulation of the subtype B_PANDEMIC_ clade. This may explain the massive influx of subtype B_PANDEMIC_ strains and the apparent absence of “non-pandemic” subtype B Caribbean lineages in Cuba.

Similarly to subtype B, there were multiple introductions of subtype C (*n*≥10), subtype G (*n*≥8) and CRF18_cpx (*n*≥2) viruses in Cuba, but only a few of them were able to get established and disseminate. The clades C_CU-I_, G_CU_ and CRF18_CU_ comprise 69%, 74% and 98% of all subtype C, subtype G and CRF18_cpx sequences from Cuba included in this study, respectively. The monophyletic clustering of CRF19_cpx-like *pol* Cuban sequences within subtype D radiation, the paucity of this genetic variant in Africa, and the recent T_MRCA_ of Cuban sequences strongly suggests that the CRF19_CU_ clade also derives from a single founder event. HIV-1 clades G_CU_, CRF18_CU_ and CRF19_CU_ probably originate in central Africa, whereas clade C_CU-I_ probably derives from east Africa. Our study suggests that clades CRF19_CU_ and G_CU_ began to circulate in Cuba around the late 1980s, followed shortly thereafter by clades CRF18_CU_ and C_CU-I_. Thus, although Cuban personnel were stationed in several African countries since the 1970s, HIV-1 African strains were successfully disseminated within Cuba only from the late 1980s onwards.

Our data suggest that HIV-1 CRFs_BG (20_BG, 23_BG and 24_BG) started to spread in Cuba in the second half of the 1990s. Such a recent expansion of BG recombinants in Cuba is fully consistent with epidemiological data showing that in samples collected in 2003, none of the individuals harboring BG recombinants were diagnosed with HIV-1 infection earlier than 1996, and all but three were diagnosed since 2000 [20]. Similarly, the proportion of BG infections among MSM in Havana City increased from 0% in those diagnosed in 1998 to 31% in those diagnosed in 2003 [3]. It was proposed that all Cuban CRFs_BG evolved from a common BG recombinant ancestor locally generated by recombination between parental clades B_CU-II_ and G_CU_ [20]. According to our estimations, that common BG recombinant ancestor was generated in the early 1990s, thus around or immediately after the estimated onset date of parental clades B_CU-II_ and G_CU_ and some years earlier than the emergence of the CRFs_BG.

The reconstruction of the demographic history indicates that most HIV-1 Cuban clades followed a very similar growth pattern characterized by rapid dissemination until the early 2000s after which the epidemic growth rate of those epidemics started to slow-down. The expansion of the B_CU-II_ clade, by contrast, seems to decrease during 1990s; whereas the growth rate of the CRF24_BG clade probably only stabilized in the second half of the 2000s. The initial expansion of the major HIV-1 Cuban clades coincides with a sustained increase in the number of infected HIV-positive individuals in Cuba from 1991 to 2000 [41]. UNAIDS estimations indicate that the total number of people living with HIV in Cuba continued to growth in the last decade, rising from 3,100 (2,600–4,300) in 2000 to 14,000 (12,000–16,000) in 2011 [36]. Our demographic analysis, however, suggests a trend toward stability in the effective number of infections of all major HIV-1 Cuban clades over time consistent with recent epidemiological data that shows a decrease of HIV incidence in Cuba, mainly among men, in the biennium 2010–2011 [42].

Our coalescent-based analyses suggest that CRF20_BG, CRF24_BG and B_CU-II_ have displayed a more explosive initial growth (1.0 year^−1^–1.6 year-^1^) than clades G_CU_, CRF18_CU_ and CRF19_CU_ (∼0.4–0.6 year^−1^); whereas clades B_CU-I_ and C_CU-I_ displayed intermediate initial growth rates (∼0.8 year^−1^). Notably, all those HIV-1 Cuban clades with the fastest initial expansion rates (B_CU-I_, B_CU-II_, B_CU-I_ and CRFs_BG) were much more prevalent among MSM than among heterosexual (HET) persons [3]. Thus, some HIV-1 Cuban clades may have spread faster than others because they encountered, by chance, local transmission chains with higher rates of partner exchange. Dissemination within a transmission network of small size and high rate of partner exchange may also explain the fast, but self limited, dissemination phase of clade B_CU-II_. Additional influence of virological factors cannot be excluded. These results must also be interpreted with caution as most growth rate estimates here obtained displayed quite large overlapping 95% HPD intervals.

It has been proposed that Cuba's low rate of HIV infection is due to several factors that served to prevent sexual transmission of the virus, including: wide-scale HIV screening and subsequent contact tracing of HIV-positive individuals, mandatory quarantine of the first HIV-infected individuals at sanatoria, free access to a well structured public health system, comprehensive HIV education campaigns, coordinated work of Cuban government agencies and community, and restricted tourism between Cuba and western countries up to the early 1990s [43], [44], [45], [46]. The estimated initial growth rates of the major HIV-1 Cuban clades (∼0.4–1.6 year^−1^), however, were comparable to those obtained for different HIV-1 epidemics in the Americas (∼0.5–1.3 year^−1^) [38], [47], [48], [49], [50], [51], [52], Europe (∼0.4–1.5 year^−1^) [52], [53], [54], [55], Africa (∼0.2–0.8 year^−1^) [47], [52], [56], [57], [58] and Asia (∼0.8 year^−1^) [59]. This suggests that several factors may have contributed to delay the introduction and/or dissemination of HIV-1 in Cuba for many years; but once some HIV-1 strains got established in vulnerable HET and MSM transmission groups they spread quickly.

In summary, this study indicates that only a few subtype B and non-B subtype founder viral strains were successfully disseminated in Cuba. Some of those HIV-1 viral strains were probably introduced from North America/Europe, central Africa and east Africa between the middle 1980s and the middle 1990s; whereas other were locally generated around the late 1990s. Changes in the social and economic landscapes of Cuba occurring at the beginning of the 1990s may have fueled the introduction and/or initial dissemination of major HIV-1 Cuban clades. Although the main HIV-1 Cuban lineages began to circulate at a rather late time of the AIDS pandemic, further dissemination within vulnerable groups was rapid. These results reinforce the importance of maintaining, reviewing and updating permanently the public health measures aimed at controlling the spread of those HIV-1 variants already established in the Cuban population.

## Supporting Information

Table S1 **HIV-1 subtype B dataset.**
(PDF)

Table S2 **HIV-1 subtype C dataset.**
(PDF)

Table S3 **HIV-1 subtype G dataset.**
(PDF)

Table S4 **HIV-1 CRF18_cpx and CRF19_cpx/subtype D datasets.**
(PDF)

Table S5 **HIV-1 CRF20/23/24_cpx datasets.**
(PDF)

Table S6 **Nucleotide substitution models selected using jModeltest program.**
(PDF)
