# Supplementary material for: Phylodynamics of the HIV-1 Epidemic in Cuba
Source: PLoS One. 2013 Sep 9;8(9):e72448. doi: 10.1371/journal.pone.0072448 (PMC3767668; doi:10.1371/journal.pone.0072448)
Supplement: Table S1 — HIV-1 subtype B dataset. (PDF) [file pone.0072448.s001.pdf]

Table S1. HIV-1 subtype B dataset.

Region	Country	*N*	Sampling date
North America	United States	525	1982-2010
Europe	France	348	1983-2008
Caribbean	Antigua and Barbuda	7	2000
	Bahamas	14	2004
	Cuba	322	1999-2011
	Dominica	3	2000
	Dominican Republic	143	2005-2010
	Grenada	4	2000
	Guyana	6	2000
	Haiti	16	2004-2005
	Jamaica	146	2001-2010
	Montserrat	1	2000
	Saint Lucia	4	2000
	Saint Vincent and The Grenadines	6	2000
	Suriname	5	2000
	Trinidad and Tobago	62	2000-2003
