# Supplementary material for: Phylodynamics of the HIV-1 Epidemic in Cuba
Source: PLoS One. 2013 Sep 9;8(9):e72448. doi: 10.1371/journal.pone.0072448 (PMC3767668; doi:10.1371/journal.pone.0072448)
Supplement: Table S2 — HIV-1 subtype C dataset. (PDF) [file pone.0072448.s002.pdf]

Table S2. HIV-1 subtype C dataset.

Region	Country	*N*	Sampling date
Caribbean	Cuba	49	2003-2011
Central Africa	Angola	31	2001-2010
	Democratic Republic of Congo	22	2002-2007
East Africa	Burundi	91	2002
	Ethiopia	81	1986-2003
	Kenya	39	1991-2007
	Tanzania	81	1997-2009
	Uganda	38	1990-2010*
Southern Africa	Botswana	70	2001
	Malawi	46	2002
	Mozambique	101	2002-2004
	Zambia	150	1998-2008
	Zimbabwe	178	2007

* Sampling date was not available for some sequences.
