# Supplementary material for: Phylodynamics of the HIV-1 Epidemic in Cuba
Source: PLoS One. 2013 Sep 9;8(9):e72448. doi: 10.1371/journal.pone.0072448 (PMC3767668; doi:10.1371/journal.pone.0072448)
Supplement: Table S3 — HIV-1 subtype G dataset. (PDF) [file pone.0072448.s003.pdf]

Table S3. HIV-1 subtype G dataset.

Region	Country	*N*	Sampling date
Caribbean	Cuba	35	1999-2011
Central Africa	Angola	13	1997-2010
	Democratic Republic of Congo	10	2002-2007
	Cameroon	37	1997-2011*
	Congo	4	-
	Equatorial Guinea	1	2008
	Gabon	6	2000-2008
West Africa	Benin	15	2004-2009
	Cape Verde	11	2005
	Ghana	6	2002-2007
	Nigeria	309	1992-2010*
	Senegal	12	2001-2010
	Togo	13	2006-2008

* Sampling date was not available for some sequences.
