# Supplementary material for: Phylodynamics of the HIV-1 Epidemic in Cuba
Source: PLoS One. 2013 Sep 9;8(9):e72448. doi: 10.1371/journal.pone.0072448 (PMC3767668; doi:10.1371/journal.pone.0072448)
Supplement: Table S4 — HIV-1 CRF18_cpx and CRF19_cpx/subtype D datasets. (PDF) [file pone.0072448.s004.pdf]

Table S4. HIV-1 CRF18_cpx and CRF19_cpx/subtype D datasets.

Subtype	Region	Country	*N*	Sampling date
CRF18_cpx	Caribbean	Cuba	62	1999-2011
	South America	Brazil	1	2005
	Europe	Spain	1	2005
		Belgium	1	1999
	Central Africa	Angola	5	2001-2010
		Cameroon	4	1997-2001*
		Congo	1	-
	Southern Africa	South Africa	1	2007
	West Africa	Nigeria	1	2008
CRF19_cpx	Caribbean	Cuba	160	1999-2011
	Europe	Greece	1	2004
		Spain	1	2002
		United Kingdom	1	2009
D	East Africa	Burundi	2	2002
		Ethiopia	1	2003
		Kenya	71	1996-2011*
		Rwanda	5	2005-2008
		Tanzania	66	2001-2009
		Uganda	872	1991-2011*
	Central Africa	Cameroon	40	1993-2010*
		Congo	7	-
		Democratic Republic of Congo	21	1983-2007
		Central African Republic	1	2009
		Gabon	5	1997-2000*
	Southern Africa	South Africa	8	1984-2008*
	West Africa	Nigeria	5	2007*
		Senegal	8	1990-2009

* Sampling date was not available for some sequences.
