# Supplementary material for: Phylodynamics of the HIV-1 Epidemic in Cuba
Source: PLoS One. 2013 Sep 9;8(9):e72448. doi: 10.1371/journal.pone.0072448 (PMC3767668; doi:10.1371/journal.pone.0072448)
Supplement: Table S5 — HIV-1 CRF20/23/24_cpx datasets. (PDF) [file pone.0072448.s005.pdf]

Table S5. HIV-1 CRF20/23/24_cpx datasets.

Subtype	Region	Country	*N*	Sampling date
CRF20_cpx	Caribbean	Cuba	62	1999-2011
	Europe	Spain	4	1999-2007
CRF23_cpx	Caribbean	Cuba	11	1999-2011
CRF24_cpx	Caribbean	Cuba	47	2010-2011
	Europe	Spain	3	2004-2008
