# Supplementary material for: Phylodynamics of the HIV-1 Epidemic in Cuba
Source: PLoS One. 2013 Sep 9;8(9):e72448. doi: 10.1371/journal.pone.0072448 (PMC3767668; doi:10.1371/journal.pone.0072448)
Supplement: Table S6 — Nucleotide substitution models selected using jModeltest program. (PDF) [file pone.0072448.s006.pdf]

Table S6. Nucleotide substitution models selected using jModeltest program.

**Dataset**	**Model**
B (CUBA + REFERENCES)	GTR+I+G
C (CUBA + REFERENCES)	GTR+I+G
G (CUBA + REFERENCES)	GTR+I+G
CRF18_cpx (CUBA + REFERENCES)	GTR+I+G
CRF19_cpx (CUBA + REFERENCES)	GTR+I+G
CRFs_BG (CUBA + REFERENCES)	TPM1uf+I+G
B_{CU-I}	TPM1uf+I+G
B_{CU-II}	TrN+I+G
C_{CU-I}	TPM1uf+I+G
G_{CU}	GTR+I+G
$CRF18_{CU}$	GTR+I+G
$CRF19_{CU}$	GTR+I+G
CRF20/23/24	TPM1uf+I+G
CRF20	GTR+I+G
CRF24	TPM1uf+I+G
